# A single-center retrospective analysis of autoimmune glial fibrillary acidic protein astrocytopathy with seizures in children

**DOI:** 10.3389/fneur.2025.1591835

**Published:** 2025-06-05

**Authors:** Hongjun Fang, Wenjing Hu, Zhi Jiang

**Affiliations:** Neurology Department, The Affiliated Children’s Hospital of Xiangya School of Medicine, Central South University (Hunan Children’s Hospital), Changsha, China

**Keywords:** autoimmune glial fibrillary acidic protein astrocytopathy, GFAP-IgG antibodies, seizure, children, GFAP-A

## Abstract

**Objective:**

We herein described the clinical characteristics of autoimmune glial fibrillary acidic protein astrocytopathy (GFAP-A) patients with epileptic seizures in the disease course.

**Methods:**

A single-center retrospective analysis of autoimmune GFAP-A with seizures was conducted.

**Results:**

There were 14 patients (35.9%, 14/39) with seizures among 39 pediatric GFAP-A patients, nine were boys and five were girls. Nine patients (64.3%, 9/14) manifested focal seizures, four (28.6%, 4/14) showed generalized tonic–clonic seizures, one (7.1%, 1/14) exhibited both forms, and five (35.7%, 5/14) manifested status epilepticus. In addition to seizures, clinical presentations included fever (71.4%), disorders of consciousness (71.4%), dyskinesia (42.9%), psychiatric symptoms (35.7%), headache (28.6%), and involuntary movements (28.6%). Electroencephalograms were all abnormal during the acute phase, principally presenting as focal or diffuse slow waves. During the acute phase, the control rate of epilepsy with immunotherapy was 50%, and seven patients still needed to be treated with antiseizure medication. After 2 years and 6 months to 4 years and 6 months of follow-up, we observed seven patients (50%, 7/14) with recurrence of seizures at 0.5–15 months after discharge, seven patients were treated with one or more antiseizure medications. Epileptic seizures were ultimately controlled in two patients, seizures diminished in one patient, treatment was ineffective in three patients, and one patient died.

**Conclusion:**

GFAP-A is an important cause of epileptic seizures in children and immunotherapy plays a crucial role. Several patients experienced chronic epileptic seizures after the acute phase and require long-term antiseizure medication, with a few showing refractory characteristics.

## 1 Introduction

Recent studies have identified immune factor-mediated neuroinflammation as an important etiology of epileptic seizures (1). Autoimmune encephalitis is an immune-mediated inflammatory encephalopathy in which sudden epileptic seizures are common in the acute phase. During the chronic phase, seizures can then develop into persistent autoimmune seizures (2). A meta-analysis of 3,722 antibody-positive patients with autoimmune encephalitis showed that 69.9% of patients experienced seizures during the course of their illness (3). Different types of antineuronal antibodies have been uncovered in patients with epileptic seizures and autoimmune encephalitis over the past decade, including those directed against the N-methyl-D-aspartate receptor (NMDAR), leucine-rich glioma-inactivated 1 (LGI1), and gamma-aminobutyric acid receptor (GABAR) (4), and their pathogenicity of these antineuronal surface-antigen antibodies has been documented (5–7). In recent years anti-glial fibrillary acidic protein (anti-GFAP) antibodies have been associated with autoimmune central nervous system diseases that present with epileptic seizures. The chief clinical presentations of autoimmune GFAP astrocytopathy (GFAP-A) include meningeal, brain parenchymal, or spinal cord inflammation, or a combination of these, with a prevalence rate of 0.6 per 100, 000 (8, 9). The characteristic imaging feature is linear perivascular radial enhancement in the white matter extending radially outward from the ventricles on magnetic resonance imaging (MRI) (10). Detection of GFAP-immunoglobulin G (GFAP-IgG) in cerebrospinal fluid (CSF) through a cell-based assay (CBA) and a tissue-based assay (TBA) is a biomarker of GFAP-A (11). In addition, GFAP-A principally occurs in adults, and only approximately 10% in children (12). Most cases of GFAP-A respond favorably to high-dose corticosteroids. Epileptic seizures constitute a characteristic clinical presentation of GFAP-A, and the incidence of epilepsy in GFAP-A patients is 10–20% (10, 11, 13, 14). The majority of studies only revealed the proportion of epileptic seizures in GFAP-A patients but did not describe the clinical characteristics of seizures in detail. Only two articles provided detailed reports on three patients with GFAP-A-associated epilepsy, including two cases of focal epilepsy and one case of super refractory status epilepticus (15, 16). There are few articles specifically related to the clinical characteristics of GFAP-A patients with seizures. The clinical spectrum, characterization of seizure semiology and data regarding long-term seizure outcomes remain unknown. We conducted a retrospective analysis of pediatric GFAP-A patients with epileptic seizures at our center to further understand the disease’s general, clinical, and imaging characteristics, electroencephalographic changes, and treatment and prognosis.

## 2 Study participants and methods

### 2.1 Patient information research methods

Our study participants were 14 patients with epileptic seizures among 39 autoimmune GFAP-A patients treated in the Neurology Department of Hunan Children’s Hospital from January 2015 to April 2024. The inclusion criteria were as follows: ① onset age is less than 18 years; ② patients presenting with meningitis, encephalitis, myelitis, or a combination of the above; ③ positive CSF exhibiting GFAP-IgG; ④ epileptic seizures in the disease course. The exclusion criterion was a definite diagnosis of other diseases. Status epilepticus (SE) is defined as an epileptic seizure continuing beyond a certain time (according to the ILAE 2015 criteria) or recurring within the same timeframe before the patient recovers baseline clinical status (17). This study was approved by the Ethics Committee of the Hunan Children’s Hospital. Written informed consent to participate in this study was provided by the participants’ legal guardian/next of kin. All methods were performed in accordance with the relevant guidelines and regulations.

### 2.2 Research methods

We collected children’s general data (sex, age, prodromal symptoms, initial symptoms, and clinical symptoms), epilepsy-related data (type of seizure, seizure frequency, and changes in disease), auxiliary examinations (laboratory tests, imaging, pathological tests, and neuroelectrophysiologic tests), treatment (drugs used for epilepsy, control results, encephalitis-treatment regimen, and treatment outcomes), and follow-up status. Indirect cellular immunofluorescence was performed to detect central nervous system demyelination antibodies and associated antibodies in the serum and CSF of all enrolled patients.

### 2.3 Laboratory studies

Testing for GFAP antibodies was conducted by TBA and CBA. For CBA, HEK293 cells were cotransfected with full-length human GFAP and pLV-mCherry-N. After 36 h of transfection in 96 well plate, the cells were fixed with 4% paraformaldehyde for 20 min, washed with PBS and permeabilized with 0.1% Triton X-100 in PBS for 20 min, which was ready for antibody detection. Serum diluted at 1:10 and CSF in PBS-10% goat serum and incubated on cells for 2 h at room temperature. Cells were then washed in PBS-0.1% Tween 20 for three times, incubated for 30 min with goat anti-human IgG (1:500, Thermo Scientific), washed again in PBS-0.1% Tween 20, and evaluated by immunofluorescence microscopy (DMI8, Germany). For TBA, Antibody detection was performed using an indirect immunefluorescence (IF) assay using standard monkey hippocampus and cerebellum tissue. Undiluted CSF sample was allowed to react with tissue sections on glass slides for 3 h at room temperature. Serum was diluted 1:100 before use and reacted with tissue sections on glass slides for 3 h at room temperature. After sample incubation, the slides were rinsed twice with phosphate-buffer saline before being incubated with fluorescein-conjugated goat anti-human IgG for 2 h. Finally, the slides were rinsed with phosphate-buffer and the fluorescence pattern was examined under a microscope. GFAP antibodies were reported as positive if both tests showed concordant results.

### 2.4 Statistical analysis

We used SPSS 24.0 for all analyses. Normally distributed quantitative data are presented as mean ± standard deviation, while non-normally distributed data are presented as the medians. Qualitative data are presented as the number of patients (percentage).

## 3 Results

### 3.1 Clinical presentation

#### 3.1.1 General data

Of the 39 patients with autoimmune GFAP-A, 14 (35.9%, 14/39) exhibited epileptic seizures during the disease course. The mean patient age was 6.43 ± 3.34 years (range: 1 year 4 months to 11 years), comprising nine boys and five girls. Five patients had a history of prodromal upper respiratory tract infection history (the specific pathogen was not determined, one had EB virus infection, and one experienced an intestinal EV-RNA virus infection). One patient was misdiagnosed with tuberculous meningitis during the early phase. This patient was negative for GFAP antibody within 1 week of disease onset, but blood and cerebrospinal fluid GFAP antibodies were positive at reexamination after 1 month and diagnosis was confirmed (Patient 1 in Table 1). Of the 14 patients, nine had meningoencephalitis and four had encephalomyelitis; one patient only had epileptic seizures as the presentation but was diagnosed due to head MRI abnormalities and blood and cerebrospinal fluid positivity for GFAP antibodies (Patient 2 in Table 1).

**Table 1:** Clinical features, auxiliary examinations, treatment strategies, prognosis in GFAP astrocytopathy patients with seizures.

Patient no.	Sex	Age at onset	Clinical symptoms besides epilepsy	CSF white blood cell count,/L; protein, g/L	Serum antibody titer	CSF antibody titer	MRI findings	Immunomodulatory therapy	ICU admission	mRS at admission/discharge	Response to therapy
1	Female	7y5 m	Fever, headache, disorders of consciousness, bowel or micturition disorder, dyskinesia	wbc:240; p:1.56	GFAP-IgG (0 → 1:100)	GFAP-IgG (0 → 1:32)	Brain: T2-hyperintense lesions in frontal, temporal, parietal, occipital, basal ganglia, thalamus, periventricular, hippocampus, corpus callosum, brain stem Spine: lesions in T1-L1 (Lesion disappeared after 6 months)	IVIG, IVMP	Yes	5/3	Symptoms improved
2	Male	7y1 m	Only epilepsy	wbc:6; p:0.19	GFAP-IgG (1:50)	GFAP-IgG (1:1)	Brain: T2-hyperintense lesions in right cerebellum Spine: normal	IVMP	No	1/0	Symptoms disappeared
3	Male	11y	Memory disorder	wbc:28; p:0.21	NMDAR-IgG (1:10) MOG-IgG (1:100)	GFAP-IgG(1:32) NMDAR-IgG(1:1) MOG-IgG(1:100)	Brain: T2-hyperintense lesions in frontal, temporal, parietal, occipital, optic nerve Spine: normal (Lesion disappeared after 2 months)	IVIG, IVMP	No	3/0	Symptoms disappeared
4	Male	1y4 m	Fever, disorders of consciousness, dyskinesia	wbc:50; p:0.6	Antibody (−)	GFAP-IgG (1:32)	Brain: T2-hyperintense lesions in frontal, temporal, parietal, occipital, cerebellum, corpus callosum, brain stem Spine: NA	IVMP	Yes	5/5	Dead
5	Female	5y8m	Fever, headache, psychiatric symptoms	wbc:0; p:0.191	GFAP-IgG (1:320)	GFAP-IgG (1:32)	Brain: T2-hyperintense lesions in right frontal, left periventricular Spine: normal	IVIG, IVMP	Yes	4/1	Symptoms improved
6	Male	3y9m	Fever, disorders of consciousness, peripheral facial palsy, impaired hearing, dyskinesia	wbc:400; p:0.63	GFAP-IgG (1:32)	GFAP-IgG (1:100)	Brain: T2-hyperintense lesions in frontal, temporal, parietal, occipital, basal ganglia, thalamus, periventricular, corpus callosum Spine: normal	IVMP	Yes	5/3	Symptoms improved
7	Female	1y10m	Fever, disorders of consciousness	wbc:233; p:0.46	GFAP-IgG (1:32)	GFAP-IgG (1:32)	Brain: T2-hyperintense lesions in thalamus, brain stem, cerebellum, left frontal Spine: lesions in C3–C6 (lesion disappeared after 2 months)	IVIG, IVMP	No	3/0	Symptoms disappeared
8	Male	6y6m	Fever	wbc:26; p:0.189	GFAP-IgG (1:32)	GFAP-IgG (1:32)	Brain: normal Spine: normal	IVMP	No	3/0	Symptoms disappeared
9	Male	10y9m	Fever, headache, disorders of consciousness, bowel or micturition disorder, dyskinesia, automatic nervous disorder, peripheral motor or sensory nerve damage	wbc:382; p:0.84	Antibody (−)	GFAP-IgG (1:32)	Brain: T2-hyperintense lesions in basal ganglia, caudate nucleus, thalamus, brain stem, cerebellum Spine: extensive lesions	IVIG, IVMP, PLEX	Yes	5/4	Symptoms improved
10	Female	9y3m	disorders of consciousness, psychiatric symptoms, sleep disorder, involuntary movements	wbc:120; p:0.21	GFAP-IgG (1:32) NMDAR-IgG (1:10)	GFAP-IgG (1:32) NMDAR-IgG (1:30)	Brain: normal Spine: NA	IVIG, IVMP	No	5/4	Symptoms improved
11	Male	10y6m	Fever, headache, disorders of consciousness, psychiatric symptoms, involuntary movements	wbc:8; p:0.19	GFAP-IgG (1:100)	GFAP-IgG (1:32)	Brain: T2-hyperintense lesions in parietal, occipital, corpus callosum Spine: NA (lesion disappeared after 2 years and 3 months)	IVIG, IVMP	Yes	5/2	Symptoms disappeared
12	Male	1y8m	Fever, disorders of consciousness, dyskinesia, peripheral motor or sensory nerve damage	wbc:80; p:0.53	GFAP-IgG (1:10)	GFAP-IgG(1:1)	Brain: T2-hyperintense lesions in frontal, temporal, parietal, occipital, thalamus Spine: normal	IVIG, IVMP	Yes	5/4	Symptoms improved
13	Male	7y6m	Fever, disorders of consciousness, dyskinesia, psychiatric symptoms, involuntary movements	wbc:2; p:0.05	GFAP-IgG (1:32)	GFAP-IgG(1:32)	Brain: T2-hyperintense lesions in frontal, temporal, parietal, occipital, periventricular Spine: normal	IVMP	Yes	5/5	Symptoms improved
14	Female	5y9m	Fever, disorders of consciousness, psychiatric symptoms, involuntary movements, bulbar palsy	wbc:14; p:0.1	GFAP-IgG (1:320)	GFAP-IgG (1:1)	Brain: T2-hyperintense lesions in frontal, parietal, occipital Spine: NA	IVIG, IVMP	Yes	5/3	Symptoms improved

#### 3.1.2 Epileptic seizure characteristics in initial course of disease

All patients developed epileptic seizures during the initial disease course, of whom seven (50%, 7/14) manifested epileptic seizures as the initial symptom. During hospitalization, nine (64.3%, 9/14) patients exhibited focal motor seizures. Of these, impaired awareness was present in five, awareness was unimpaired in two. Other seizure semiologies included generalized tonic–clonic seizures in four patients (28.6%, 4/14), and one (7.1%, 1/14) manifested both generalized tonic–clonic seizures and focal motor seizures with impaired awareness. During the initial disease course, epileptic seizures occurred only once in two (14.3%, 2/14) patients, twice in one (7.1%, 1/14) patient, and recurrently in the remaining 11 (78.6%, 11/14) patients. The duration of epileptic seizures varied: 11 (78.6%, 11/14) patients experienced epileptic seizures lasting for less than 5 min and three (21.4%, 3/14) patients had epileptic seizures lasting for 5–10 min. Among these patients, five (35.7%, 5/14) exhibited status epilepticus, with the longest lasting 2 h (Table 2).

**Table 2:** Seizures characteristics, EEG findings, antiseizure medications strategies, prognosis in GFAP astrocytopathy patients with seizures.

	During admission						Follow up after discharge after discharge					
Patient no.	Days from symptom onset to first seizure	Type of seizure (seizure frequency)	SE	Interictal EEG	Seizures captured on EEG	ASMs	Total follow-up duration	Time from discharge to recurrent seizures	Type of seizure (seizure frequency)	SE	ASMs	Response to therapy
1	1w	Focal (1 episode)	No	Slow waves in the right brain region +spike wave	No	No	3y8m	15 m	Focal (1–2 episodes/m)	No	LCS, PER	Failure
2	1d	Focal (1episode/2-7d)	No	Spike wave	No	OXC	2y1 m	0.5 m	Focal (4–5 episodes/ m)	No	OXC	Failure
3	1d	Focal (1–4 episodes/d)	No	Slow waves in the right parietal and temporal regions	No	No	3y1 m	No seizure	/	/	No	/
4	2d	GTCS (several episodes/4 d)	Yes	Severe and widespread low voltage	No	LEV	4y1 m	1 m	Focal (several episodes/w)	No	LEV	Dead
5	1d	Focal (1–4 episodes/ d)	No	Slow waves in the left central, parietal, and occipital regions	Three focal seizures	LEV, LCS	1y8m	4 m	Focal (9 episodes/4 m)	No	LCS, LEV	Seizure-free
6	2 W	Focal (2 episodes)	No	Diffuse slow waves	No	No	3y4 m	No seizure	/	/	No	/
7	1d	Focal (1 episode)	No	Diffuse slow waves	No	No	3y	No seizure	/	/	No	/
8	1d	GTCS+Focal (1–3 episodes/ d)	Yes	Slow waves in the right temporal region	One BIRDs, one electrical seizures	OXC	1y4 m	2 m	Focal (3 episodes/3d)	No	OXC + IVMP	Seizure-free
9	1 W	GTCS (several episodes/ w)	Yes	Diffuse slow waves	No	No	2y6m	No seizure	/	/	No	/
10	1d	Focal (2–4 episodes/ d)	Yes	Slow waves in the left brain region	No	No	4y	no seizure	/	/	No	/
11	4d	Focal (1–4 episodes/1–2 d)	No	Diffuse slow wave	Five electrical seizures	LEV, CZP	3y11 m	0.5 m	Focal (several episodes/m)	No	LEV+OXC + VPA + IVMP	Failure
12	5d	GTCS(3 episodes)	No	Diffuse slow wave	No	No	4y6m	No seizure	/	/	No	/
13	1d	GTCS (1–7 episodes/d)	Yes	Diffuse slow wave+spike wave	No	LEV	3y4 m	No seizure	/	/	LEV	/
14	4d	Focal (1–3 episodes/d)	No	Diffuse slow waves+sharp waves in the posterior brain region	One NCSE	LEV, VPA	2y	5 m	Focal (1 episode/ several months)	No	VPA + PER	Reduction

CSF, cerebrospinal fluid; EEG, electroencephalography; MRI, magnetic resonance imaging; mRS, modified Rankin Scale; IVIG, intravenous immunoglobulin; IVMP, intravenous methylprednisolone; PLEX, Plasma exchange; MOG, myelin oligodendrocyte glycoprotein; NMDAR, N-methyl-d-aspartate receptor; NA, no available; ASMs, antiseizure medications; SE, status epilepticus; GTCS, generalized tonic–clonic seizure; BIRD, brief potentially ictal rhythmic discharge; NCSE, non-convulsive status epilepticus; VPA, valproate; LEV, levetiracetam; OXC, oxcarbazepine; LCS, lacosamide; CZP, clonazepam; y, year; m, month; d, day; WBC, white blood cell; P, protein.

#### 3.1.3 Clinical presentations other than epileptic seizures

In addition to epileptic seizures, other clinical symptoms during the course of the disease included fever (*n* = 10), disorders of consciousness (*n* = 10), dyskinesia (*n* = 6), psychiatric symptoms (*n* = 5), headache (*n* = 4), involuntary movements (*n* = 4), peripheral motor or sensory nerve damage (*n* = 2), bowel or micturition disorder (*n* = 2), bulbar palsy (*n* = 1), memory disorder (*n* = 1), sleep disorder (*n* = 1), peripheral facial palsy (*n* = 1), automatic nervous disorder (*n* = 1), and impaired hearing (*n* = 1). Nine of these children were admitted to the ICU due to severe condition and five received ventilator support due to respiratory failure. Twelve patients developed complications, including seven patients with pneumonia (two with severe pneumonia), five with electrolyte disturbance, four with impaired hepatic function, two with intracranial hypertension, two with severe sepsis, two with myocardial injury, one with cerebral hemorrhage, and one with urinary tract infection. Ten (71.4%) patients had a modified Rankin scale (mRS) score of 4–5 points and four patients (28.6%) had an mRS score of 1–3 points at disease peak; the median mRS score was 5 (3, 5) points (Table 3).

**Table 3:** Summary of clinical presentation and associated conditions in GFAP astrocytopathy patients with seizures.

Items	Incidence
Feature	
Males:females	9:5
Mean age (y)	6.43 ± 3.34
Intensive care unit	9/14 (64.3%)
Main symptoms	
Fever	10/14 (71.4%)
Disorders of consciousness	10/14 (71.4%)
Dyskinesia	6/14 (42.9%)
Psychiatric symptoms	5/14 (35.7%)
Headache	4/14 (28.6%)
Involuntary movements	4/14 (28.6%)
Peripheral motor or sensory nerve damage	2/14 (14.3%)
Bowel or micturition disorder	2/14 (14.3%)
Seizure characteristics during the acute phase	
Focal seizures	9/14 (64.3%)
GTCS	4/14 (28.6%)
Both GTCS and focal seizures	1/14 (7.1%)
SE	5/14 (35.7%)
Epileptic seizures as the initial symptom	7/14 (50.0%)
EEG findings	
Abnormal	14/14 (100.0%)
Severe low voltage	1/14 (7.1%)
Focal slow waves	5/14 (35.7%)
Diffuse slow wave	7/14 (50.0%)
Epileptiform discharge	4/14 (28.6%)
Neuroimaging	
Brain	12/14 (85.7%)
Spinal cord	3/14 (21.4%)
Optic nerve	1/14 (7.1%)
Enhancement	8/14 (57.1%)
CSF abnormality	
Elevated protein	5/14 (35.7%)
Elevated leukocyte	9/14 (64.3%)
Multiple antibodies positive	2/14 (14.3%)
Immunotherapy during the acute phase	
IVMP	5/14 (35.7%)
IVMP+IVIG	8/14 (57.1%)
IVMP+IVIG+PLEX	1/14 (7.1%)
Recurrent seizure characteristics after discharge	
Patients of recurrent seizures	7/14 (50.0%)
Time from discharge to recurrent seizures	0.5–15 m
focal seizures	7/7 (100.0%)
Seizure freedom achieved	2/7 (28.6%)
seizure reduction	1/7 (14.3%)
Seizure failure	3/7 (42.9%)
Dead	1/7 (14.3%)

### 3.2 Laboratory tests

**Figure 1:**
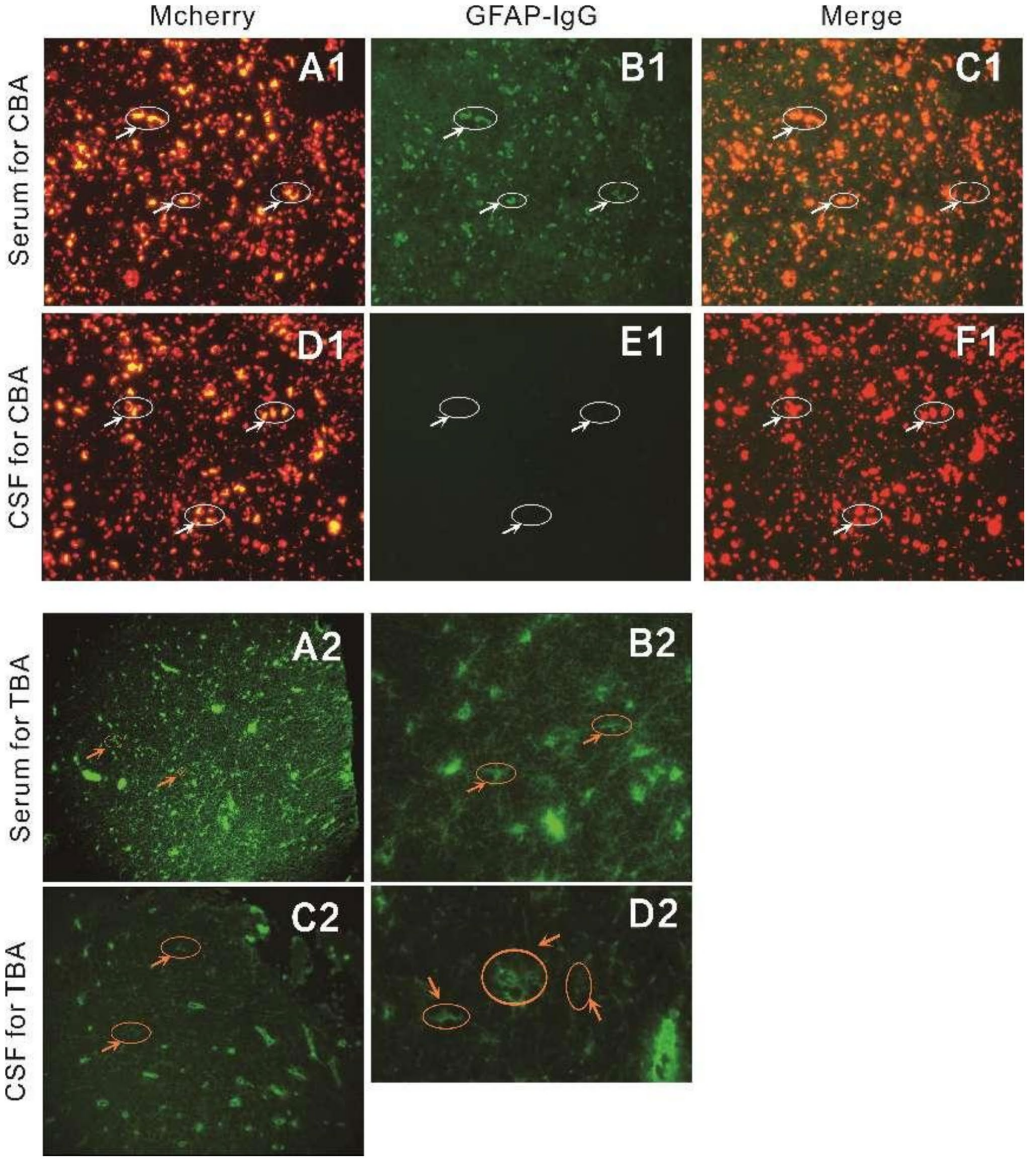
Detection of anti-GFAP antibody by CBA **(A1–F1)** and TBA **(A2–D2)** in patient 14. **(A1–F1)** GFAP-IgG were tested by a CBA using HEK293 cells transiently cotransfected with full-length human GFAP and pLV-mCherry-N. The patient’s IgG bound to GFAP-transfected cells and showed green fluorescence as a positive control. **(A1–C1)** GFAP-IgG in serum were tested. **(D1–F1)** GFAP-IgG in CSF were tested. **(A2–D2)** TBA was performed with an indirect immunofluorescence assay using standard monkey hippocampus and cerebellum tissue. **(A2–B2)** Serum specific IgG binds to the white matter astrocytes in monkey cerebellar tissue, consistent with GFAP distribution. **(C2–D2)** CSF specific IgG binds to the white matter astrocytes in monkey cerebellar tissue, consistent with GFAP distribution.

Routine cerebrospinal fluid biochemical tests were completed in the acute phase in all 14 pediatric patients, with nine showing elevated cerebrospinal fluid white blood cell count (normal values, 0–20 × 10^6^/L; range, 26–400 × 10^6^/L). Among the patients, three, one, and five had white blood cell count of 20–50 × 10^6^/L, 50–100 × 10^6^/L, and >100 × 10^6^/L, respectively. Five patients exhibited elevated cerebrospinal fluid protein levels, ranging from 0.53 to 1.56 g/L. Cytologic test results were principally monocyte and lymphocyte elevations, and neutrophils and activated monocytes were observed in several patients. Eleven of the 14 patients tested positive for both blood and cerebrospinal fluid GFAP antibodies, while three patients were only positive for antibodies in the cerebrospinal fluid (Figure 1). One child’s fluid reflected overlapping NMDA antibody and one showed both overlapping NMDA antibody and myelin oligodendrocyte glycoprotein (MOG) antibody.

### 3.3 Electroencephalogram

**Figure 2:**
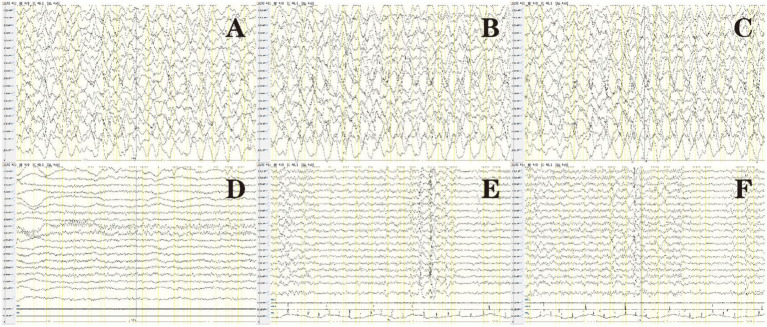
Electroencephalogram of patient 14 in Table 1: before treatment **(A–C)**, after treatment **(D)**, and after seizure recurrence **(E,F)**. **(A)** Background diffuse slow waves. **(B)** Spike waves were primarily present in the bilateral occipital and posterior temporal regions. **(C)** Eye opening in a patient, poor response to external stimuli, and absence of convulsions. Synchronized electroencephalography showed diffuse high-extremely high wave amplitude in various brain regions and the absence of intermittent discharge at the 1–1.5 Hz *δ* area, indicating NCSE. **(D)** Normal electroencephalography after treatment. **(E,F)** After epileptic seizures recurred, electroencephalograms showed right frontal pole, frontal, and anterior temporal spike waves, and several discharges of spike-slow waves were observed during sleep.

All patients underwent at least one video electroencephalography during the initial disease onset, and the monitoring duration was 4–15 h. All patients had abnormal electroencephalographic results: one with severe low voltage, one with epileptiform discharge but no slowing, five with focal slow waves involving one to multiple brain regions and with epileptiform discharge simultaneously detected in one patient, and seven with diffuse slow waves, of which two showed epileptiform discharge. All study subjects had epileptic seizures, but epileptiform discharge was only detected in four patients. Epileptiform discharge involved multiple brain regions, of which the temporal region (*n* = 4), center (*n* = 2), parietal region (*n* = 2), and frontal region (*n* = 1) were common. We detected three focal seizures, six electrical seizures, one brief potentially ictal rhythmic discharge (BIRD), and one episode of non-convulsive status epilepticus (NCSE) (Figure 2) using video electroencephalography of four patients. Focal or electrical seizures primarily originate in the central, parietal, temporal, and occipital regions. The initial electroencephalographic pattern was mainly spike waves or sharp waves, and seizures lasted for 40–115 s. Four patients demonstrated suspected epileptiform activity, which was ruled out using synchronized video electroencephalography. Abnormal activity primarily presented as positive bilateral eye rolling, frequent bilateral upward gazing, paroxysmal bilateral dazed eyes, right lower limb convulsion, upper limb lifting, forceful limb movements, strabismus, or blinking, waveforms were not detected in the seizure phase.

### 3.4 Imaging

**Figure 3:**
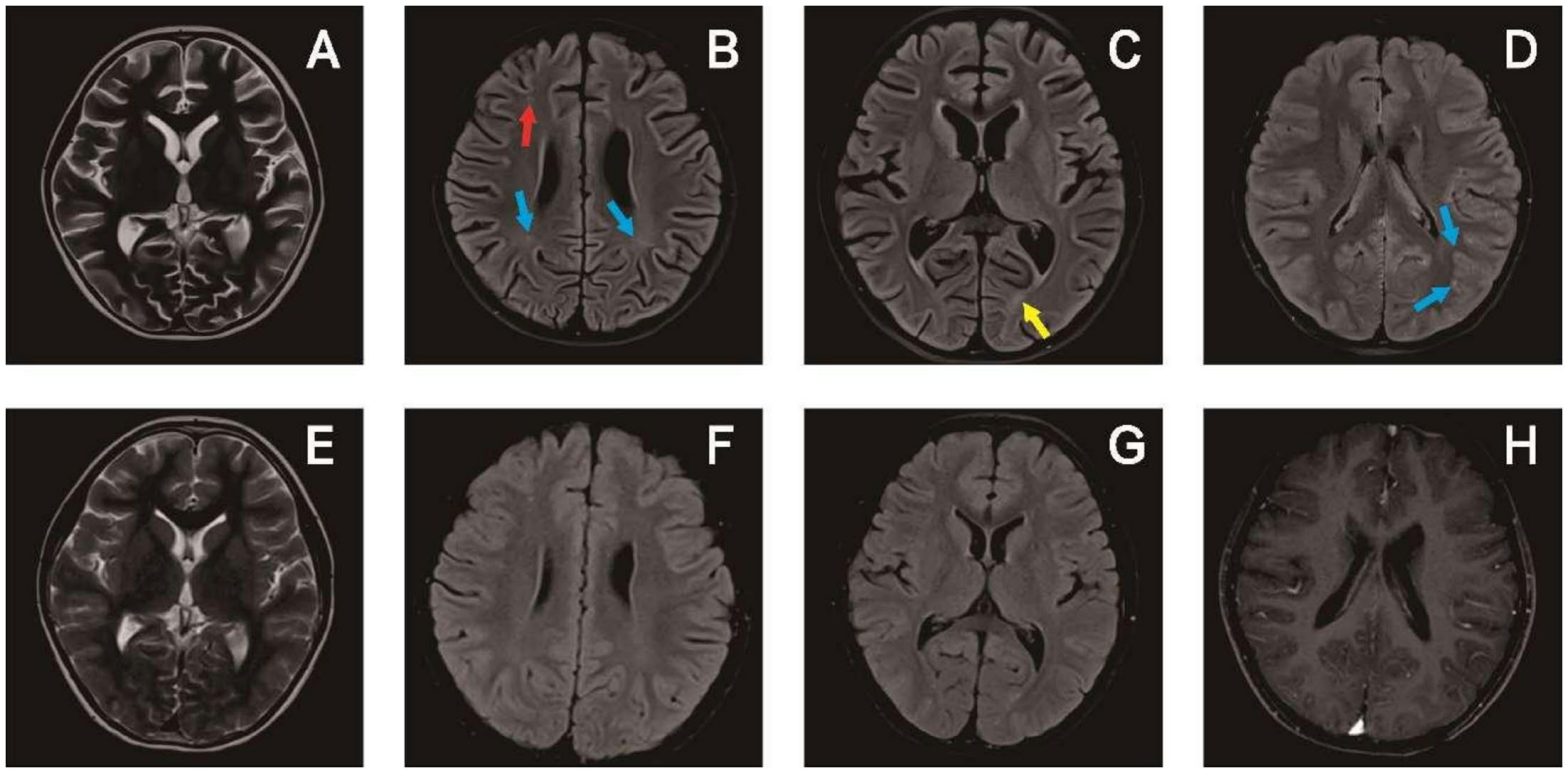
Brain MRI of patient 14 in Table 1: upon admission **(A–D)** and 1 year later **(E–H)**. **(A)** T2 image showed widening of the cerebral sulcus. **(B)** T2-hyperintense lesions in the white matter of the frontal lobe (red arrow) and parietal lobe (blue arrow). **(C)** T2-hyperintense lesions in the white matter of the occipital lobe (yellow arrow). **(D)** Enhancement of T2 flair showed small strip like high signal shadows in the sulci of the brain. **(E–H)** Follow-up images of improved T2 lesions and enhancement.

**Figure 4:**
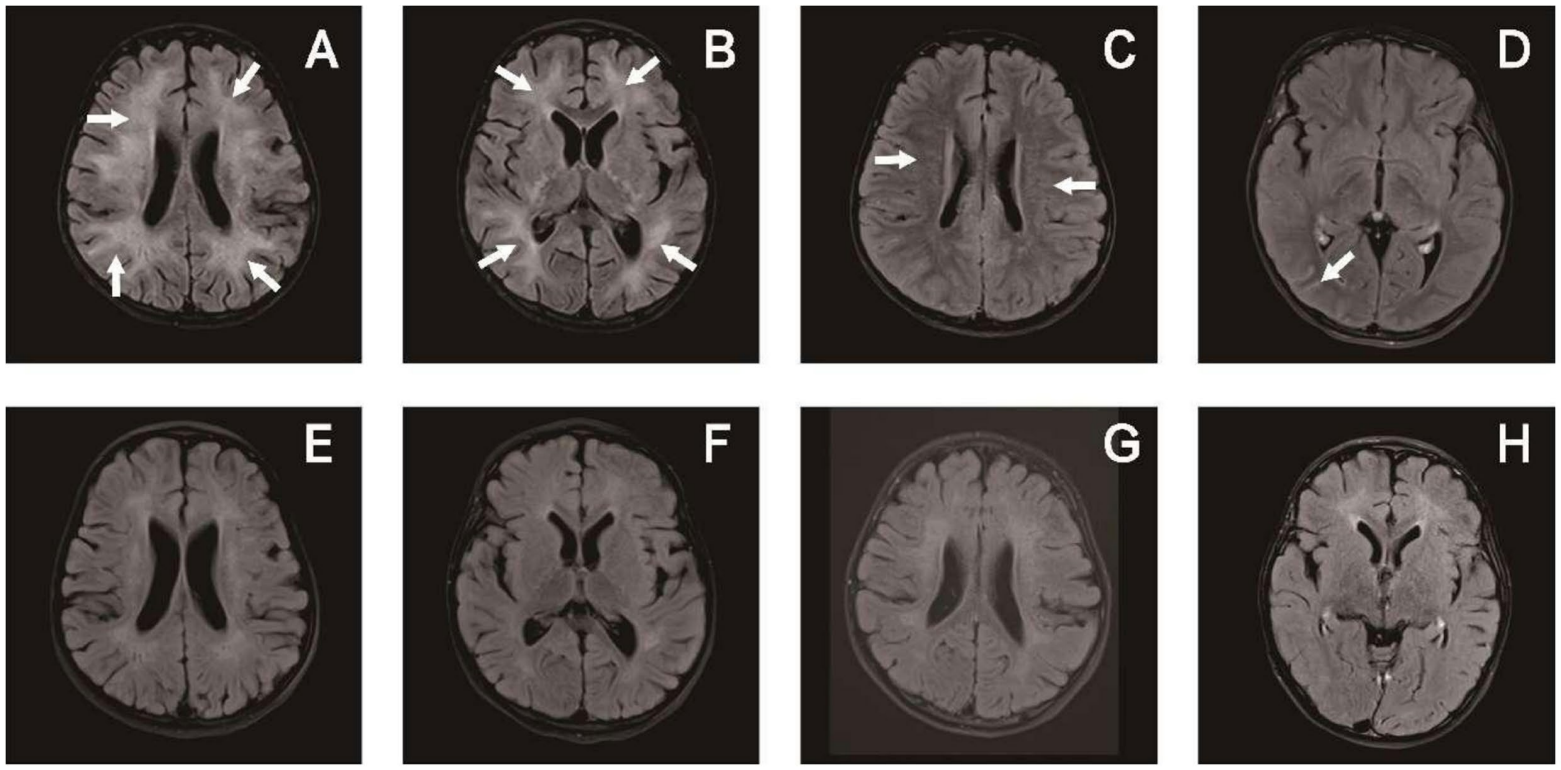
Brain MRI of patient 6 in Table 1: upon admission **(A–D)** and 3 months later **(E–H)**. **(A,B)** T2-hyperintense lesions in the white matter of bilateral cerebral hemispheres. **(C)** Periventricular radial linear enhancement. **(D)** Enhanced shadow of occipital lobe sulci. **(E–H)** Follow-up images of improved T2 lesions and enhancement.

A head MRI scan was completed in 14 patients and a spinal cord MRI scan was completed in 10 patients. Eight of these scans showed abnormalities on head MRI, three reflected head and spinal cord MRI abnormalities, and one manifested head and optic nerve MRI abnormalities. Eight patients also exhibited enhanced shadows. The heads of the children chiefly showed cortical and subcortical white matter involvement, with 1–10 sites of involvement. Bilateral lesions were the most common (*n* = 8) and all were asymmetric, numbering twice that of the unilateral lesions (*n* = 4). The involved sites included the frontal lobe (*n* = 9), parietal lobe (*n* = 8), occipital lobe (*n* = 8), temporal lobe (*n* = 6), thalamus (*n* = 5), periventricular white matter (*n* = 4), cerebellum (*n* = 4), brain stem (*n* = 3), basal ganglia (*n* = 3), corpus callosum (*n* = 3), caudate nucleus (*n* = 1) and hippocampus (*n* = 1). Only three patients presented with spinal cord lesions: one comprised thoracic-lumbar spinal cord T1-L1, one involved the cervical spinal cord C3-C6, and one encompassed extensive spinal cord lesions. Lesions diminished or disappeared after treatment in the 11 patients with imaging abnormalities, and four children had complete disappearance of the lesion on MRI reexamination, occurring 2 months to 2 years and 3 months after disease onset (Figures 3, 4).

### 3.5 Treatment protocol

#### 3.5.1 Immunotherapy

Five patients underwent methylprednisolone pulse therapy (10–20 mg/kg, 3–5 days), and nine patients received methylprednisolone pulse therapy and intravenous immunoglobulin (IVIG), one of whom underwent plasmapheresis before IVIG. All patients received oral prednisone for maintenance therapy.

#### 3.5.2 Antiseizure medication

During their first hospitalization, 14 patients were treated with immunotherapy, and seven of them had acute seizure control. The control rate of epilepsy with immunotherapy was 50%, and seven patients still needed to be treated with antiseizure medication, four of whom received monotherapy and three received combination therapy. Monotherapy comprised oxcarbazepine (*n* = 2) and levetiracetam (LEV, *n* = 2), and combination therapy comprised LEV + lacosamide (LCM, *n* = 1), LEV + clonazepam (CZP, *n* = 1), and LEV + sodium valproate (VPA, *n* = 1). Five patients received diazepam and midazolam as temporary antispasmodic treatments due to frequent convulsions or status epilepticus.

### 3.6 Determination of response and follow-up

After discharge, six of the 14 patients achieved favorable outcomes, while eight (57.1%) had poor outcomes, including residual cognitive disability, movement disorder, and convulsions. Post-discharge outpatient or telephone follow-up was conducted and the mean follow-up duration was 3.04 ± 0.98 years. After discharge, seven patients did not develop epileptic seizures after 6 months to 4 years and 6 months of follow-up, and the mean follow-up duration was 2.68 ± 1.16 years. The remaining seven patients (50.0%) developed epileptic seizures that occurred 0.5–15 months after discharge, with a median duration of 2 (0.5, 5) months. While these seven patients all showed focal seizures after discharge, they did not develop status epilepticus. All pediatric patients who developed convulsions after discharge received antiseizure medications: two patients received monotherapy and five received combination therapy, of whom two underwent combined pulse steroid therapy. Of the seven patients, epileptic seizures were controlled in two patients (including one patient receiving repeated immunotherapy) and seizures did not recur after more than 1 year, one patient experienced a reduction in seizures, three patients (including one patient receiving repeated immunotherapy) showed low antiseizure medication effectiveness and still had several seizures each month, and the remaining patient died owing to recurrent convulsions and a secondary lung infection after discharge.

## 4 Discussion

Autoimmune GFAP-A is a relatively new autoimmune disease of the central nervous system that was first named by Fang et al. (18) from the Mayo Clinic, USA, in 2016; the authors reported that an IgG that specifically targeted GFAP was present in experimental animals and patients with this disease. The chief clinical presentation of autoimmune GFAP astrocytopathy (GFAP-A) includes meningeal, brain parenchymal, or spinal cord inflammation, or a combination of these. Although studies have shown that epileptic seizure are a clinical presentation of GFAP-A (19), only two small-scale case series (a total of three cases) have provided detailed descriptions of epileptic seizures in GFAP-A (15, 16). Most studies have only revealed the proportion of patients with GFAP-A who experienced seizures, without detailing the clinical features of seizures. Therefore, little is known about the characteristics of epileptic seizures in GFAP-A, especially in pediatric patients. In our study, we reported 14 patients (35.9%) who presented with seizures among 39 pediatric patients with GFAP-A. To the best of our knowledge, this is the largest clinical study of GFAP-A patients with seizures.

In the present study, 35.9% of GFAP-A patients developed epileptic seizures during the disease course (with significantly more males than females), and our proportion of patients was significantly higher than that of pediatric and adult GFAP-A patients with epileptic seizures (10–20%), as reported in the literature (10, 11, 13, 14). With the exception of one patient with a clinical presentation of epileptic seizures alone, the other patients presented with encephalopathy, primarily meningoencephalitis and encephalomyelitis. GFAP-A exhibits diverse types of epileptic seizures. During the acute phase, it mainly presents as focal seizures, followed by generalized tonic–clonic seizures; however, after the acute phase, 50.0% of patients redevelop epileptic seizures that present as focal seizures. Most studies have revealed that epileptic seizures are present in patients with GFAP-A, but these did not describe the type of seizure in detail. These investigators ascertained that five patients (35.7%, 5/14) developed status epilepticus in the early phase of the disease, which was generally consistent with the proportion (6–40%) of NMDAR encephalitis patients with status epilepticus (20). Table 2 shows that among five patients with status epilepticus during the early phase of the disease, only two (40%, 2/5) experienced epileptic seizures during the post-discharge follow-up, and five of nine patients without status epilepticus (55.6%, 5/9) developed epileptic seizures again during follow-up. This finding supports the concept that status epilepticus in the early phase of the disease cannot be used as an indicator of subsequent autoimmune encephalitis-related epilepsy. In addition to epileptic seizures, the other clinical presentations in our 14 patients were fever, disorders of consciousness, dyskinesia, psychiatric symptoms, headache, and involuntary movements, which are generally consistent with previous reports (21). During the peak of disease, 10 of our patients (71.4%) had an mRS score of 4–5 points, nine patients (64.3%) were admitted to the ICU due to a severe condition, and five (35.7%) patients were provided ventilator support due to respiratory failure; these rates were all higher than the mean levels for GFAP-A patients (21, 22). This indicates that epileptic seizures in patients with GFAP-A may be associated with initial disease severity.

In our study, 12 of 14 patients showed imaging abnormalities of the head and two had normal imaging presentations. During follow-up, one of these two patients with a normal head MRI developed epileptic seizures again, which were ultimately controlled after antiseizure medication. Of the remaining 12 patients with head MRI abnormalities, head lesions on MRI completely disappeared 2 months to 2 years and 3 months after the disease onset. Of these four patients, two had epileptic seizures again during follow-up and antiseizure medication effectiveness was low in these patients. This shows that epileptic seizures caused by imaging abnormalities of the head are associated with poor outcome. We asked, ‘What is the pathogenesis of epilepsy in patients with a normal imaging presentation?’ Studies have shown that astrocyte activation increases the risk of epilepsy and astrocyte proliferation and that increased astrocyte GFAP expression is associated with the severity of epilepsy (23, 24). These microscopic astrocyte changes may explain why epilepsy occurs in GFAP-A with a normal imaging presentation.

In the present analysis, 100% of pediatric patients showed abnormal electroencephalograms, which was higher than the head MRI abnormality rate and mainly presented as diffuse or focal slow waves. Epileptiform discharge was detected in several patients, and this was for the most part consistent with the majority of immune encephalitis cases (25, 26). Herein, we noted only one patient with extensive severe low voltage during the early phase of the disease. Although electroencephalography findings improved after treatment, the patient died due to recurrent convulsions and secondary lung infection after discharge. Thus, a low voltage in electroencephalograms may be associated with a poor prognosis. Involuntary movements are also present in patients with GFAP-A, and it is sometimes difficult to distinguish these from epileptic seizures based on clinical presentation alone. In the current study, we determined the clinical and subclinical seizures in four patients. Thus, of the 14 patients, four had suspected epileptiform activity that was finally ruled out through synchronized video electroencephalography. We recognize that electroencephalography plays an important role in distinguishing epileptic events from non-epileptic events in patients with GFAP-A, and that subclinical electrical seizures can be detected in an effort to guide treatment.

Two of our patients exhibited overlap syndrome, one manifesting overlapping NMDA antibodies and one with both overlapping NMDA and MOG antibodies. A recent meta-analysis revealed that the probability of acute epileptic seizures in patients with MOG antibody disease is generally 20.5% (27). Another analysis showed that approximately 70% of anti-NMDAR encephalitis patients developed epileptic seizures (28, 29). We then asked, ‘What epileptogenic mechanisms are present in GFAP overlap-syndrome patients, and which antibodies reflect a dominant role?’ However, the specific mechanisms underlying these effects remain unclear. One study showed that the simultaneous presence of GFAP antibody and other well-characterized antibodies (such as NMDAR and MOG) in autoimmune overlap syndrome was due to elevated astrocyte activation or destruction, exposing the GFAP antigen and increasing GFAP immune responses. Thus, the production of GFAP antibodies may be critical for the pathogenicity of GFAP-A (30, 31). Another study speculated that immune reconstitution during the tapering of immunosuppressive drugs might activate production of new autoantibodies; therefore, special attention should be paid to slow tapering of steroids (32). Overlapping autoantibodies are common in GFAP astrocytopathy, involving MOG-IgG, NMDAR-IgG, or other neuronal antibodies. The exact difference between GFAP-A patients with overlapping and non overlapping syndromes is still unclear. The system screening patients with GFAP-A for other antibodies is helpful in understanding patient’s condition.

In this study, all patients received pulse steroid therapy: nine patients experienced combined gamma globulin therapy, one patient underwent additional plasmapheresis, and several patients received antiseizure medication. There are disparities in therapeutic strategies for GFAP-A. Currently, there is no treatment standard or consensus, and it is still unclear whether long-term antiseizure medication is required for epileptic seizures. Some investigators have indicated good responses to corticosteroids, and the majority of their patients showed clinical improvement after immunotherapy, including decreased acute epileptic seizures (30, 33). Therefore, acute long-term antiseizure medication is not recommended. In the present study, seven patients developed epileptic seizures again during the post-discharge follow-up, and these patients received long-term antiseizure medication after epileptic seizures recurred. Only two patients (patients 8 and 11) underwent repeated combined pulse steroid therapy. Patient 8 received steroids combined with antiseizure medication after convulsions, with no convulsions occurring 1 year after treatment. However, patient 11 still manifested recurrent convulsions after administration of steroids combined with antiseizure medication. The limitations of the current study include the difficulty in determining the prophylactic effects of immunosuppressants combined with antiseizure medication in patients who develop epileptic seizures after their condition has stabilized. Sriram et al. (16) reported a female patient diagnosed with GFAP-A-associated super-refractory status epilepticus, which was initiated on intravenous methylprednisolone (IVMP) but showed no improvement. She subsequently underwent plasma exchange (PLEX) and showed a reduction in number. She was administered intravenous immunoglobulin (IVIG), bortezomib, rituximab, and tocilizumab. She showed gradual yet significant improvement and was ultimately seizure-free. Therefore, it is crucial to initiate early and aggressive immunotherapy with multiple agents that target various parts of the autoimmune pathway to successfully manage the disease. The sample size of this study was small, and the follow-up duration of some patients was short. Therefore, a cohort study with a larger sample size and longer duration is required to explore this area further.

In summary, epileptic seizures were common in pediatric patients with GFAP-A, with most male patients, and the most common type of seizure was focal in our study. Head MRI chiefly showed cortical and subcortical white matter involvement, and electroencephalograms primarily exhibited focal and diffuse slow waves. Acute glucocorticoid and/or immunoglobulin treatment can also be used to control the disease. Several of our patients demonstrated secondary epileptic seizures after the acute phase and required long-term antiseizure medication or immunotherapy, while several patients had refractory disease. We posit that electroencephalography and imaging are helpful for patients with early acute epileptic seizures or isolated epileptic seizures without any cause and recommend that GFAP antibody testing be carried out as soon as possible to determine the diagnosis.

## Data Availability

The original contributions presented in the study are included in the article/supplementary material, further inquiries can be directed to the corresponding author.
